# Influence of Distinct Maternal Cytomegalovirus-Specific Neutralizing and Fc Receptor-Binding Responses on Congenital Cytomegalovirus Transmission in HIV-Exposed Neonates

**DOI:** 10.3390/v17030325

**Published:** 2025-02-26

**Authors:** Itzayana G. Miller, Aakash Mahant Mahant, Jennifer A. Jenks, Eleanor C. Semmes, Eric Rochat, Savannah L. Herbek, Caroline Andy, Nicole S. Rodgers, Justin Pollara, Linda M. Gerber, Betsy C. Herold, Sallie R. Permar

**Affiliations:** 1 Department of Pediatrics, Weill Cornell Medicine, New York, NY 10065, USA; igm4001@med.cornell.edu (I.G.M.); savannahherbek@gmail.com (S.L.H.); 2 Immunology and Microbial Pathogenesis Program, Weill Cornell Medicine Graduate School of Medical Sciences, New York, NY 10065, USA; 3 Department of Microbiology-Immunology, Albert Einstein College of Medicine, Bronx, NY 10461, USA; aakash.mahant@einsteinmed.edu (A.M.M.); betsy.herold@einsteinmed.edu (B.C.H.); 4 Human Vaccine Institute, Duke University, Durham, NC 27710, USA; jenjenks@stanford.edu (J.A.J.); eleanor.semmes@childrens.harvard.edu (E.C.S.); ejrochat@gmail.com (E.R.); jpollara@duke.edu (J.P.); 5 Medical Scientist Training Program, Department of Molecular Genetics and Microbiology, Duke University, Durham, NC 27710, USA; 6 Department of Population Health Sciences, Weill Cornell Medicine, New York, NY 10065, USA; cma2008@med.cornell.edu (C.A.); lig2002@med.cornell.edu (L.M.G.); 7 Department of Surgery, Duke University School of Medicine, Durham, NC 27710, USA; nicole.rodgers@duke.edu; 8 Department of Pediatrics, Children’s Hospital at Montefiore, Bronx, NY 10461, USA

**Keywords:** cytomegalovirus, HCMV, humoral immunity

## Abstract

Congenital cytomegalovirus (cCMV) is the most common infectious cause of birth defects worldwide, affecting approximately 1 in every 200 live-born infants globally. Recent work has identified potential immune correlates of protection against cCMV transmission including maternal and placentally transferred antibody levels and their function, which may inform the development of maternal active (vaccine) and passive (mono/polyclonal antibody) immunizations. However, these correlates need to also be assessed in diverse cohorts, including women living with HIV who have increased risk of cCMV transmission. Using a case–control design, we investigated whether the magnitude, specificity, function and placental transfer of maternal IgG responses are associated with protection against and/or risk of cCMV transmission in HIV/HCMV co-infection. Within 3 historical cohorts of pregnant women with HIV/HCMV co-infection, we identified 16 cCMV transmitting cases that were matched to 29 cCMV non-transmitting controls. Using a systems serology approach, we found that normalized HCMV-specific IgG binding to FcγR1α was higher in non-transmitting dyads, whereas HCMV-neutralizing antibody responses were higher in transmitting dyads. These findings suggest that engagement of FcγR1α by HCMV-specific IgG may help confer protection against cCMV transmission. Building upon previous research, our study reinforces the critical role of validating maternal humoral immune correlates of cCMV transmission risk across diverse seropositive cohorts, providing essential insights to inform and accelerate the development of effective HCMV vaccines.

## 1. Introduction

Congenital human cytomegalovirus (HCMV) circulates widely within the human population and infection is typically asymptomatic or only mildly symptomatic for immunocompetent individuals [1]. However, HCMV can cause severe fetal disease if congenitally transmitted, which occurs both in pregnant individuals who have pre-existing immunity and those with a primary infection in pregnancy [2]. Congenital cytomegalovirus (cCMV) is the most common infectious cause of birth defects worldwide, affecting at least 1 in every 200 live-born infants (0.5%) globally [3,4,5], and affecting pregnancies at a rate ranging from 0.6% to up to 5%, in part influenced by the overall seroprevalence of HCMV in the regional population [6]. Varying sequelae result from cCMV infection, ranging from clinically inapparent in the majority of infants, to sensorineural hearing loss and severe neuropathology in up to 25% of all congenitally infected children [7,8].

The higher cCMV transmission rates after primary viral infection (30–40%) compared to viral re-infection (3.4%) and reactivation (less than 1%) indicate a partially protective role of the adaptive maternal immune system against cCMV transmission [3,7,9,10]. Leveraging the maternal immune system through vaccination remains an important strategy to prevent cCMV. However, there are no currently licensed vaccines to prevent HCMV acquisition or placental transmission. The most successful vaccine to date is the gB/MF59 vaccine, a soluble, postfusion gB subunit protein that conferred approximately 50% protection against primary HCMV acquisition [11,12]. IgG binding to cell-associated gB was identified as a correlate of protection in CMV seronegative women receiving this vaccination [13]. A number of HCMV vaccine candidates are currently in development, using vaccine platforms such as disabled infectious single cycle [14], recombinant subunit [15] and the mRNA/LNP [16] which is currently in phase 3 trials in seronegative women. Although the risk for cCMV is highest if primary CMV is acquired during pregnancy, the majority of cCMV transmission globally occurs in HCMV seropositive pregnant individuals, and thus, an HCMV vaccine should address cCMV transmission in both seronegative and seropositive populations. Humoral immune responses that have been identified as associated with decreased risk of cCMV transmission include high maternal plasma pp150-specific IgG levels in early gestation [17], high-avidity IgG binding to HCMV, antibody-dependent cellular phagocytosis (ADCP) [18] and antibody-dependent cellular cytotoxicity (ADCC) [19]. Yet, the immune correlates of protection against vertical CMV transmission in seropositive pregnant women should be validated in diverse cohorts to facilitate the development and evaluation of candidate vaccines for the prevention of cCMV.

Neonates born to pregnant women living with HIV (WLWH) are at particularly high risk for cCMV acquisition, with transmission rates ranging from 1.5% to 11%, rates which may be modified by maternal ART treatment status [1,6,20]. The increased risk of cCMV transmission in the setting of untreated HIV infection has been attributed, in part, to impaired maternal T cell immunity, more frequent CMV reactivation and higher CMV viral loads, but whether antibody responses contribute to risk for transmission has not been well studied. Maternal HIV infection is associated with dysfunctional B cell responses, hypergammaglobulinemia and impaired placental IgG transfer [21,22]. To investigate whether the magnitude, specificity, function and placental transfer of maternal HCMV-specific IgG responses are associated with protection against and/or risk of cCMV transmission in WLWH, we identified 16 cCMV transmissions (cases) from the following 3 historical cohorts of HCMV seropositive pregnant WLWH recruited internationally from 1997 to 2013: (i) NICHD International Site Development Initiative (NISDI. Perinatal study), (ii) Pediatric AIDS Clinical Trials Group (PACTG, study 316) and (iii) The International Maternal Pediatric Adolescent AIDS Clinical Trials Group (IMPAACT, study P1025). Cases were matched 1:2 to HIV/HCMV coinfected non-transmitting dyads (*n* = 29) based on maternal age, gravida, parity and CD4 T cell counts. Using a systems serology approach, we identified seven distinct HCMV-specific IgG measurements as potential correlates of risk or protection against cCMV transmission in these cohorts. Elucidating the humoral immune correlates of protection against cCMV in the high risk setting of maternal HIV and HCMV co-infection will address the gap in knowledge of protective immunologic responses against cCMV across diverse populations, a gap that is currently impeding HCMV vaccine development for this important demographic in the global cCMV epidemic.

## 2. Materials and Methods

### 2.1. Study Population

We studied biobanked samples from 3 historical cohorts, Pediatric AIDS Clinical Trials Group (PACTG, protocol 316, *n* = 33), International Maternal Pediatric Adolescent AIDS Clinical Trials Group (IMPAACT, perinatal core protocol 316, *n* = 6) Network and NICHD International Site Development Initiative (NISDI, Perinatal protocol, *n* = 6). Cases of cCMV transmission were previously identified in PACTG (Protocol 316, *n* = 11) and IMPAACT (Perinatal core protocol, *n* = 2) via neonate plasma or PMBC qPCR [23]. We performed a similar HCMV immediate early (IE-1) gene-based qPCR screen of 396 neonate plasma samples collected at the earliest available timepoint (median 3, average 3.14, range 0–64 days of age) from the NISDI Perinatal cohort that had not previously been screened for cCMV infection (Supplementary Table S1). Our screen identified an additional 2 cases of cCMV transmission for a total of 15 cCMV transmissions (cases). Cases were matched by propensity score at a 1:2 ratio to HIV/HCMV coinfected non-transmitting dyads (*n* = 30) based on maternal age (>25 or <25), race (Black, White, Asian, not specified), ethnicity (Hispanic, non-Hispanic or not specified), gravida (number or births), parity (nulliparous or non-nulliparous), CD4 counts (>200 or <200 cells/) and gestational age. Confirmatory qPCR (see HCMV qPCR) was run for neonate samples. Exemption from human subject research was obtained from Duke University’s Institutional Review Board (21-0523559, 21-07023713).

### 2.2. HCMV qPCR

NICHD International Site Development Initiative neonate plasma was screened for HCMV DNAemia by qPCR to identify cases of cCMV as previously described [18]. Additionally, by the same method, we confirmed cCMV transmission status from Pediatric AIDS Clinical Trials Group (PACTG, *n* = 33) and International Maternal Pediatric Adolescent AIDS Clinical Trials Group (IMPAACT *n* = 6) Network samples were confirmed transmission status. Briefly, 105–200 uL of plasma was ultracentrifuged (3 h at 30,000× *g*). A total of 20 uL of plasma including the virus pellet was frozen at −80 °C. Viral DNA from the virus pellet was isolated with QIAmp DNA kit (Qiagen, 51304) and tested via quantitative PCR (qPCR) in quadruplicate using SYBR Select (ThermoFisher, Waltham, MA, USA, 4472908) and 200 nM forward (5′-CAAGCGGCCTCTGATAACCA-3′) and reverse (5′-ACTAGGAGAGCAGACTCTCAGAGGAT-3′) primers to amplify the highly conserved HCMV immediate early-1 (IE1) gene. Samples were analyzed on the QuantStudio 6 flex Real-Time PCR system. Cycle threshold values were compared to an HCMV IE1 plasmid standard (six-point serial dilution starting at 10 × 10^9^ copies/well run in duplicate). HCMV DNA concentration was interpolated using the absolute quantification (standard curve method) with a standard curve ran on each plate to determine sample plasma HCMV viral load [24]. Vertical transmission was defined as infant plasma that had detectable CMV in 2 or more of 4 technical replicates above the 250 copies/mL limit of detection for each sample.

### 2.3. IgM ELISA

Detection of HCMV specific IgM was conducted via ELISA using a commercially available kit per manufacturer instructions (BioRad, Hercules, CA, USA, 25178). Optical density (OD) was read on the Biotek Synergy LX microplate reader (Agilent, Santa Clara, CA, USA). Index value was determined for all samples with the single calibrator method as described by the manufacturer.

### 2.4. Total IgG Levels

Maternal and neonate plasma total IgG levels were determined as previously described using an in-house ELISA [25]. Briefly, ELISA plates were coated with 2 ug/mL goat anti-human Ig (Invitrogen, Waltham, MA, USA, H1700). Eight-point plasma serial dilutions starting at 1:1000 with 4-fold serial dilutions were used in duplicate with HCMV-hyperimmunoglobulin (Cytogam) and anti-RSV monoclonal antibody (Synagis) of known concentration used as positive controls. Blank wells served as negative controls. Human IgG standard (Sigma Aldrich, St. Louis, MI, USA, I2511) was used to interpolate plasma IgG levels using a 5-parameter standard curve. Peroxidase AffiniPure Goat Anti-Human IgG, Fcγ fragment specific (Jackson ImmunoResearch, West Grove, PA, USA, 109-035-098) at a 1:10,000 dilution was used, followed by SureBlue Reserve TMB substrate (KPL, 5120-0083; 20 μL/well). Reactions were stopped by Stop Solution (KPL, 5150-0021; 20 μL/well) after 5 min and optical density (OD) was detected at 450 nm (Biotek, Winooski, VT, USA, Synergy LX). Duplicates with CVs > 25% were repeated. Total IgG transfer efficiency was calculated as (neonate IgG)/(maternal IgG) × 100%.

### 2.5. Cell Culture and HCMV Virus Production

Human retinal pigment epithelial (ARPE-19) cells (ATCC, CRL-2302) were maintained in DMEM supplemented with 10% FBS and Pen-Strep. Human foreskin fibroblast HFF-1 cells (ATCC, SCRC-1041) were maintained in DMEM supplemented with 20% FBS, 2 mM L-glutamine, 25 mM HEPES, Pen-Strep and 50 μg/mL Gentamicin. Human monocytes THP-1 cells (ATCC, TIB-202) were maintained in Roswell Park Memorial Institute Medium (RPMI) 1640 media (ThermoFisher, 12633012) supplemented with 10% fetal bovine serum (Sigma Aldrich, F4135). For all tissue culture, cells were maintained in 5% CO_2_ at 37 °C. HCMV virus strains Toledo and AD169r were produced as previously described [18]. Briefly, T-175 flasks with confluent HFF-1 cells were infected at an MOI of 0.01. After 14 days, cells were harvested and lysed by multiple freeze–thaw cycles. Cell-associated supernatant and cell-free supernatant were filtered (0.45 μm), then concentrated on a 20% sucrose cushion (1.5 h at 53,000× *g*) in a Beckman SW28 rotor. Virus titer was determined by the limited dilution technique in 96 well plates.

### 2.6. Whole-Virion HCMV IgG Binding and Avidity

IgG binding and avidity were assessed as previously described [18]. Briefly, ELISA plates were coated with Toledo (250 PFU) and AD169r (500 PFU) per well. Eight-point plasma serial dilutions starting at 1:30 with 3-fold serial dilutions were used in duplicate. HCMV-hyperimmunoglobulin (Cytogam) and anti-RSV monoclonal antibody (Synagis) were used as positive and negative controls, respectively. Wells were washed with either PBS or 7M urea (VWR international, 57-13-6). Peroxidase AffiniPure Goat Anti-Human IgG, Fcγ fragment specific (Jackson ImmunoResearch, 109-035-098) at a 1:5000 dilution was used, followed by SureBlue Reserve TMB substrate (KPL, 5120-0083; 20 μL/well). Reactions were stopped by Stop Solution (KPL,5150-0021; 20 μL/well) after 10 min and optical density (OD) was detected at 450 nm (Biotek, Synergy LX). Cytogam was used as a standard to interpolate plasma Cytogam equivalent IgG levels using a 5-parameter standard curve. Duplicates with CVs > 25% were repeated. Virus specific IgG transfer efficiency was calculated as (neonate IgG)/(maternal IgG). Relative Avidity Index (RAI) within a single sample was calculated as (urea IgG)/(PBS IgG) × 100% for samples within Cytogam linear range.

### 2.7. HCMV IgG1 and IgG3 Binding

The IgG1 and IgG3 subclass binding against AD169r virus strain was determined by ELISA. Plates were coated with AD169r virions (2000 PFU/well). Eight-point plasma serial dilutions starting at 1:10 with 5-fold serial dilutions were used in duplicate. Cytogam and Synagis were used as positive and negative controls, respectively. Horseradish peroxidase (HRP)-conjugated anti-Human IgG1 Fc (ThermoFisher, MH1715) or mouse anti-Human IgG3 hinge (ThermoFishsher, 05-3620) at a 1:250 dilution was used, followed by SureBlue Reserve TMB substrate (KPL, 5120-0083; 100 μL/well). Reactions were stopped by Stop Solution (KPL, 5150-0021; 100 μL/well) after 25 min and optical density (OD) was detected at 450 nm (Biotek, Synergy LX). Duplicates with CVs > 30% were repeated. AUC was calculated using GraphPad Prism (v9).

### 2.8. Binding Antibody Multiplex Assay (BAMA) Levels and Avidity

BAMA was run as previously described [18]. HCMV, HSV and HIV proteins were coupled to fluorescent beads (Luminex). HCMV gH/gL, HCMV gH/gL/gO and HIV Con6gp120 proteins were produced in-house. HCMV gB (Sino Biological), HCMV PC (Cedarlane) are commercially available. HSV gD-1 and HSV gB were produced at the Albert Einstein College of Medicine Protein Production Facility. Plasma was diluted (1:25 for UL141, UL16 and gH/gL proteins or 1:500 for all other proteins) in diluent, plated in duplicate and then co-incubated with antigen-coupled beads then washed with either PBS (binding) or citric acid (avidity; Teknova, Hollister, CA, USA, Q2444). IgG binding was detected with mouse anti-human IgG-PE (Southern Biotech, Birmingham, AL, USA, 9040-09) and mean fluorescent intensity was measured (Bio-Plex 200). Duplicates with CVs > 25% were repeated. Transfer efficiency between dyads was calculated as (neonate MFI)/(maternal MFI) × 100%. Avidity within a single sample was calculated as (citric acid MFI)/(PBS MFI) × 100% for samples within the Cytogam linear range.

### 2.9. Fc Binding by Glycoprotein Specific IgG

As previously described [18], FcR binding by HCMV (gB, gH/gL/gO, Pentameric complex), HSV (gB, gD-1) and HIV (gp120) glycoproteins coupled fluorescent beads (Luminex) was measured using a modified BAMA. Purified FcR1α, FcR2α (clone H131), FcgR3 (clone F158) and FcRn were produced by the Duke Human Vaccine Institute (DHVI) protein production facility and biotinylated in-house. Maternal and neonate plasma was diluted (1:500) in diluent, plated in duplicate and then co-incubated with antigen-coupled beads. Next, biotinylated FcRs were complexed with Streptavidin-PE (Southern Biotech, 7105-09L), then co-incubated with antibody bound beads after washing. Mean fluorescent intensity was measured (Bio-Plex 200). Duplicates with CVs > 25% were repeated, if high CV persisted, average of 4 replicates is reported. Transfer efficiency between dyads was calculated as (neonate MFI)/(maternal MFI) × 100%. Normalized of FcR binding to total glycoprotein specific binding was calculated as (glycoprotein FcR MFI)/(glycoprotein MFI).

### 2.10. HCMV Neutralization Assay

The 50% neutralizing titer (ID50) was determined against 2 strains of HCMV as described previously [18,26]. Plasma starting at 1:30 with 3-fold serial dilutions were co-incubated with whole-HCMV virions for 1 h at 37 °C. HCMV strain AD169r was tested with both ARPE-19 (MOI 1) and HFF-1 (MOI 2) and HCMV strain Toledo (MOI 0.5) with HFF-1 cells only. To quantify HCMV infection, plates were first fixed with 10% formalin, then stained with mouse anti–HCMV IE1 gene (1:500; Millipore, MAB810), followed by goat anti–mouse IgG–AF488 (1:500; Abcam, Cambridge, UK, ab150113), and cell nuclei were stained with DAPI (1:1000; Thermo Fisher, 62248). Cytogam and Synagis were used as positive and negative controls, respectively. Total cells and AF488^+^ cells were counted on an ImageXpress Pico Automated Cell Imaging System (Molecular Devices). Duplicates with CVs > 25% were repeated. Subsequently, % infection rate was determined by the AF488^+^ cells/total cells in each well and neutralization titers (ID_50_) was calculated as the sample dilution that caused a 50% reduction in the number of infected cells compared with wells treated with virus only.

### 2.11. Whole-HCMV Virus Phagocytosis

Antibody-dependent cellular phagocytosis (ADCP) was measured by flow cytometry as previously described [18,26]. Briefly, a total of 2.0 × 10^5^ PFU of AD169r or 4.0 × 10^5^ PFU of Toledo virions were conjugated to 10 µg of AF647 N-hydroxysuccinimide ester (Invitrogen, A37573). Fluorescently labeled virus was diluted to 50 PFU/uL (AD169r) or 100 PFU/uL (Toledo) in wash buffer. A total of 500 PFU (AD169r) or 1000 PFU (Toledo) were used per well. In a 96-well plate, fluorescently labeled virus was co-incubated with maternal and neonate plasma (1:60) at 37 °C for 2 h to allow immune complex formation before adding 50,000 THP-1 cells per well. A serial dilution of Cytogam was included as a positive control, while seronegative sera samples and Synagis were included as negative controls. Plates were then centrifuged (1200× *g*) at 4 °C for 1 h in a spinoculation step before a 1 h incubation at 37 °C to allow for phagocytosis. Next, cells were washed and fixed with 1% Formalin. Events were acquired on an BD LSR Fortessa flow cytometer and the percentage of AF647-positive cells was calculated from the live THP-1 monocyte parent population using FlowJo. Unstained cells and single-color stained cells were included as controls for setting gates and compensation. Fluorescence was measured using a BD Fortessa flow cytometer, and data are reported as the percent AF647^+^ live population. The cut-off for positivity was the mean signal of HCMV seronegative samples plus 3 standard deviations and duplicates with CVs > 25% were repeated.

### 2.12. Natural Killer (NK) Cell CD107a Degranulation ADCC Assay

NK cell degranulation was quantified by surface expression of CD107a as previously described [19]. Briefly, HCMV AD169r infected MRC-5 fibroblasts (MOI = 1 or mock infected), were co-incubated with primary human NK cells isolated from PBMCs (human NK cell isolation kit; Miltenyi Biotec) of a healthy adult donor. Live NK cells were added to each well containing HCMV-infected or mock-infected fibroblasts at an effector/target (E:T) ratio of 1:1. Cytogam IgG product or diluted sera samples (1:75) were then added with brefeldin A (GolgiPlug; BD Biosciences, Milpitas, CA, USA), monensin (GolgiStop; BD Biosciences) and anti–CD107a FITC (clone H4A3; BD Biosciences). After a 6 h incubation, NK cells were stained with anti–CD56-PE/Cy7 (clone NCAM16.2; BD Biosciences), anti–CD16-PacBlue (clone 3G8; BD Biosciences) and a viability dye (Live/Dead Aqua Dead Cell Stain, Thermo Fisher Scientific). Events were acquired on an LSR Fortessa flow cytometer, and the frequencies of live, CD107a+ NK cells were calculated in FlowJo (gating strategy in Supplemental Figure S3). To correct for nonspecific degranulation activity, the signal in mock-infected wells was subtracted from the signal in HCMV-infected wells for each sera sample (Supplemental Figure S3)

### 2.13. Statistical Analysis and Modeling

All primary data underwent independent quality control by at least 2 independent lab members using standardized criteria based on duplicate well variance and performance of positive and negative controls, which included Cytogam, HCMV seropositive and HCMV seronegative sera samples. Wilcoxon’s signed-rank tests were used to compare immune measures within maternal-neonate dyads, and between transmitting vs. non-transmitting groups. False discovery rate (FDR) correction for multiple comparisons was applied. Supplemental Table S5 includes all raw and FDR corrected *p*-values.

The logistic regression model sought to identify relevant features of maternal antibodies which may be associated with the transmission of cCMV from mother to infant. As this analysis is exploratory in nature, the dataset contains more variables than predictors, introducing statistical complications related to high dimensionality. As a preliminary step in dimension reduction, predictors were retained if they had a significant univariate association with CMV transmission (FDR corrected *p* < 0.05). Considering the subset of variables with significant univariate associations, variable importance was further assessed through use of four variable selection modeling procedures including stepwise automated variable selection logistic regression, LASSO, elastic net and random forest modeling. This process of performing variable selection using four methods was implemented to overcome limitations related to sample size and multicollinearity of predictors. By this approach, variables may be classified as “important” if they are retained in at least two of the four automated selection procedures listed above. A final logistic regression model predicting CMV transmission was generated containing all “important” predictors of cCMV transmission, defined as selected at least two of the four automated variable selection procedures.

## 3. Results

### 3.1. Study Population

We identified cases of cCMV (transmitting) from mother–neonate dyads who participated in 3 different clinical studies in PLWH. Thirteen cases were previously established by plasma or PBMC HCMV qPCR [23], and of these, 11 cases originated from the Pediatric AIDS Clinical Trials Group (PACTG, study 316) [27], and 2 cases originated from the International Maternal Pediatric Adolescent AIDS Clinical Trials Group (IMPAACT, study P1025) [28].

We identified additional 2 cases from the NICHD International Site Development Initiative (NISDI, Perinatal protocol) [29,30] clinical study by screening plasma collected from newborns (mean 3.14, range 0–64 days of life) with an in-house HCMV qPCR targeting the immediate early protein (IE-1) HCMV gene (Supplemental Table S1). In the first identified case of cCMV, plasma from PTID 70017A, a non-Hispanic Black or African American male, was screened at 4 days of age, resulting in 2469 copies/mL HCMV DNA. In the second identified case of cCMV, plasma from PTID 72074A, a Hispanic female, with the reported race either unknown or not reported, was screened at 2 days of age, resulting in 392 copies/mL HCMV DNA.

A total of 15 cases were matched 1:2 based on the propensity score of previously identified risk factors of cCMV transmission (maternal age, >25 or <25; race, Black, White, Asian, or not specified; ethnicity, Hispanic or non-Hispanic; gravida, parity, nulliparous or non-nulliparous; CD4+ T cell counts, >200 or <200 cells/mm^3^) to HIV/HCMV coinfected non-transmitting dyads (*n* = 30) [31,32,33]. Demographic and clinical measures were matched between maternal participants (Table 1).

All neonate samples underwent a confirmatory screen of HCMV DNAemia using the same in-house plasma HCMV qPCR against immediate early protein (IE-1) HCMV gene. Via this screen, 1 previously identified non-transmitting dyad [23] was reassigned to cCMV-transmitting classification due to a detectable HCMV viral load (385 copies/mL) at 9 days of life for a final cohort of 16 CMV-transmitting and 29 non-transmitting dyads. We did not observe significant differences in demographic characteristics between the groups. Importantly, we observed similar HIV viral loads and CD4+ T cell levels between in cCMV-transmitting compared to non-transmitting mothers (Table 1 and Supplementary Figure S1A,B). Thus, HIV-associated disease progression was similar between the groups, allowing for a focus on the associations of HCMV-specific immunity and cCMV transmission risk in the setting of maternal HIV infection [20,23].

### 3.2. Similar HCMV-Specific IgG Avidity Comparing cCMV-Transmitting and Non-Transmitting WLWH

**Figure 1:**
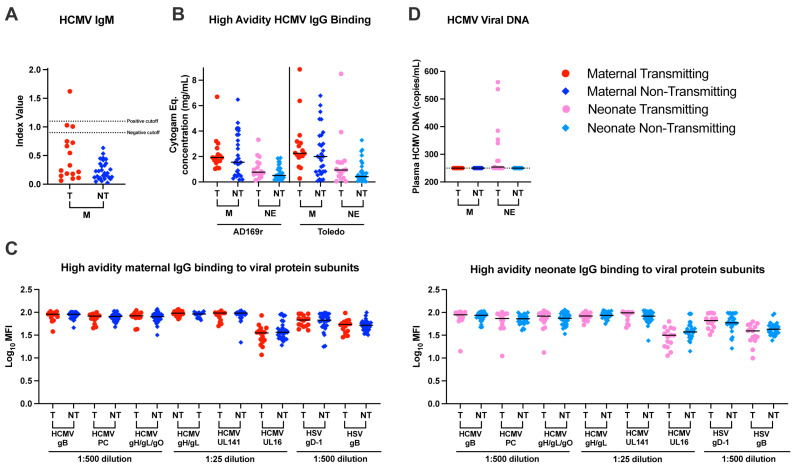
Similar HCMV-specific IgM and high-avidity IgG binding in transmitting and non-transmitting dyads. Whole virion IgM was quantified using manufacturer instructions and reported as an index value. HCMV virion IgG binding was measured using a modified ELISA including incubation with urea and reported as the Cytogam equivalent concentration. HCMV and HSV viral protein subunit specific IgG binding was measured with a modified Luminex based assay including incubation with citric acid. Samples were tested at the specified plasma dilution and reported as MFI. Urea and citric acid were used as chaotropic agents to disassociate weakly binding antibodies. Plasma HCMV DNA levels were determined by qPCR targeting HCMV IE1 using a standard curve to interpolate viral load. Maternal (M) and neonate (NE) responses were measured within and between transmitting (T) and non-transmitting (NT) dyads. (**A**) HCMV specific IgM, (**B**) High-avidity HCMV IgG binding, (**C**) High-avidity IgG binding to viral antigens in maternal (left) and neonate (right) plasma. (**D**) HCMV plasma viral load. Red circle indicates maternal transmitting group, pink circle indicates neonate transmitting group, dark blue diamond indicates maternal non-transmitting group and light blue diamond indicates neonate non-transmitting group. Horizontal black bars denote median. FDR corrected *p* values for comparisons within maternal or neonatal samples reported for Wilcoxon’s signed-rank test (**A**–**D**) with *p* < 0.05 noted numerically. No statistical tests conducted for plasma viral load.

The risk of cCMV transmission following primary infection is associated with high HCMV-specific IgM and low-avidity IgG [34], but whether these serologies are related to cCMV transmission in the setting of reinfection or reactivation is not well established. Among the cCMV-transmitting mothers, 1/15 (6%), had detectable plasma HCMV IgM compared to 0/30 (0%) non-transmitting mothers (Figure 1A). In addition, two cCMV-transmitting mothers had a plasma IgM in the equivocal range of the assay and would require a more sensitive method or later collection point to establish HCMV IgM status. HCMV-specific IgG avidity was measured using both a modified whole-HCMV virion ELISAs and multiplex assays in which an additional incubation with urea or citric acid, respectively, was used to disassociate low affinity antibodies. We observe similar levels of avidity of IgG antibodies against the whole-HCMV virion (Figure 1B), as well as HCMV and HSV surface glycoproteins (Figure 1C) between cCMV-transmitting and non-transmitting mothers. HCMV viral load did not differ between transmitting and non-transmitting mothers (Figure 1D). Taken together, the similar rate of HCMV-specific IgM detection and similar avidity IgG antibodies suggests that these serologies are not a strong predictor of risk of transmission and that most of these WLWH had not recently acquired CMV infection.

### 3.3. Higher Total and HCMV Glycoprotein-Specific IgG Responses in cCMV-Transmitting WLWH

**Figure 2:**
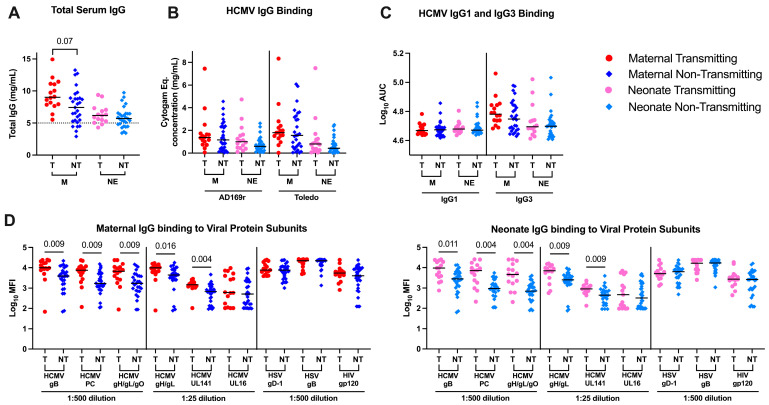
HCMV specific IgG binding in transmitting and non-transmitting maternal-neonate dyads. Total and HCMV specific antibody levels were measured by ELISA and reported as concentration, Cytogam equivalent concentration, or area under the curve (AUC). HCMV, HSV and HIV viral protein subunit specific IgG binding was measured at the specified dilution using a Luminex based assay and reported as MFI. Maternal (M) and neonate (NE) responses were measured within and between transmitting (T) and non-transmitting (NT) dyads. (**A**) Total plasma concentrations (**B**,**C**) HCMV virion IgG (**B**), IgG1 and IgG3 (**C**), (**D**) IgG binding to HCMV envelope glycoproteins in maternal (left) and neonate (right). Red circle indicates maternal transmitting group, pink circle indicates neonate transmitting group, dark blue diamond indicates maternal non-transmitting group and light blue diamond indicates neonate non-transmitting group. Dashed line in (**A**) indicates previously observed normal IgG levels in a pregnant cohort. Horizontal black bars denote median. FDR corrected *p* values for comparisons within maternal or neonatal samples reported for Wilcoxon’s signed-rank test (**A**–**D**) with *p* < 0.05 noted numerically.

High total plasma IgG levels have previously been associated with cCMV transmission risk [35,36] and untreated HIV infection is known to lead to hypergammaglobulinemia [37]. To determine whether plasma IgG levels are associated with cCMV transmission in the setting of HIV infection, we measured total plasma IgG (not HCMV-specific) concentrations by ELISA. Higher plasma total IgG concentrations were detected in cCMV-transmitting (median 9.02, range 5.51–14.93 mg/mL) compared to non-transmitting women (median 7.42, range 2.9–13.26 mg/mL), which are higher ranges than recent studies of maternal IgG levels and cCMV risk in HIV-uninfected women (median 5.27, range 1.70–7.193 mg/mL, Figure 2A) [38].

To determine if the high total plasma IgG was reflected in an increase in viral specific IgG levels in the transmitting women, we compared HCMV-specific total and subclass IgG responses against the whole-HCMV virion and individual HCMV surface glycoproteins, as well as antibodies to herpes simplex virus (HSV) and HIV by ELISA. HSV was included as another chronic herpesvirus infection, commonly associated with more frequent reactivation episodes in WLWH [39]. There were no significant differences in plasma HCMV virion-specific IgG, IgG1 or IgG3 levels comparing cCMV-transmitting and non-transmitting mothers across two HCMV strains: the attenuated AD169r laboratory strain with a repaired Pentameric Complex (PC) [40] and the Toledo clinical strain which has a deficient PC and is unable to infect epithelial cells [41] (Figure 2B,C). However, similar to previous reports in U.S. cCMV-transmitting cord blood donors [25], plasma HCMV glycoprotein-specific IgG levels were higher in cCMV-transmitting compared to non-transmitting mothers (Figure 2D right) and their neonates (Figure 2D left). This trend of higher-magnitude virus-specific IgG levels was also observed in HCMV glycoprotein-specific IgG responses (gB, PC, gH/gL, gH/gL/gO and UL141), but was not observed for HSV (gB and gD-1) or HIV (gp120) glycoprotein responses (Figure 2D). These results suggest that the high total plasma IgG in cCMV-transmitting WLWH may reflect specific antigenic responses (e.g., higher HCMV-glycoprotein specific IgG but not HSV or HIV specific IgG levels).

### 3.4. Impaired Transplacental IgG Transfer Efficiency in cCMV-Transmitting and Non-Transmitting Dyads

IgG is actively transported across the placenta by the neonatal Fc receptor (FcRn), resulting in transfer ratios (neonatal/maternal IgG levels) greater than 1. However, hypergammaglobulinemia may interfere with the transfer of antigen specific Abs by competing for the FcRn [42]. To determine whether the higher plasma IgG of cCMV transmitting vs. non-transmitting women was associated with impaired placental transport, we measured IgG in neonatal blood and calculated the placental transfer ratio. There was no difference in total IgG transplacental transfer ratios comparing cCMV-transmitting (median 0.76, range 0.33–1.25) and non-transmitting (median 0.82, range 0.38–2.03) dyads. As expected, the transfer ratio median values were less than 1, indicating a cohort-wide impaired placental IgG transfer (Supplemental Figure S2A) [43]. This observation is consistent with previous literature describing decreased transplacental IgG transfer in the setting of maternal HIV infection [44,45].

Similarly, there were no significant differences in the placental IgG transfer ratios of HCMV-specific virion or glycoproteins, HSV gD or gB, or HIV gp120 antibodies comparing cCMV transmitting vs. non-transmitting dyads. Yet, the median transfer ratios again were less than 1, indicative of cohort-wide impaired placental IgG transfer (Supplemental Figure S2B,C). Transplacental IgG transfer of high-avidity whole-HCMV virion-specific IgG was also less than 1 (Supplemental Figure S2D). These findings differ from results obtained in HIV-uninfected cohorts where, for example, the placental IgG transfer ratio of HSV gB and gD-specific IgG was 1.45 [range 1.3–2.3] and 1.35 [range 1.2–1.7], respectively, in full-term mother–neonatal dyads.

### 3.5. Higher HCMV-Neutralizing Antibodies, but Not ADCC and ADCP Function in cCMV-Transmitting Versus Non-Transmitting WLWH

**Figure 3:**
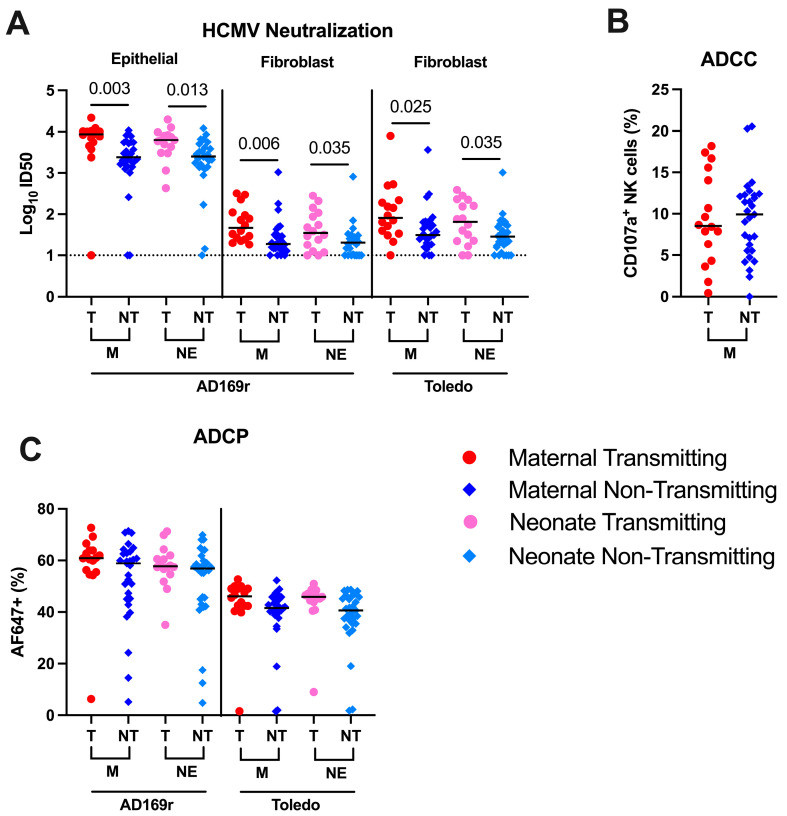
Higher HCMV-neutralizing antibodies in transmitting compared to non-transmitting groups, but not ADCC and ADCP function. Neutralizing antibody titers were measured by HCMV IE1 staining and reported as the plasma dilution that inhibiting 50% of HMCV infection (ID_50_). Antibody-dependent cellular cytotoxicity (ADCC) was assessed by NK cell degranulation at a 1:50 plasma dilution and reported as percentage of CD107a-positive NK cells. Antibody-dependent cellular phagocytosis (ADCP) of AF647-conjugated HCMV virions (AD169r or Toledo strains) by THP-1 monocytes was measured by flow cytometry and reported as percent AF647-positive cells. IC_50_, percent CD107a-positive NK cells and percent AF647-positive cells from neutralization. Responses were measured within and between transmitting (T) and non-transmitting (NT) dyads. (**A**) HCMV neutralization across virus strains (AD169r and Toledo) and cell types (fibroblast (HFF) and epithelial cell (ARPE)). (**B**) ADCC in maternal samples only, (**C**) ADCP across two HCMV virus strains (AD169r and Toledo). Red circle indicates maternal transmitting group, pink circle indicates neonate transmitting group, dark blue diamond indicates maternal non-transmitting group and light blue diamond indicates neonate non-transmitting group. Horizontal black bars denote median. FDR corrected *p* values for comparisons within maternal or neonatal samples reported for Wilcoxon’s signed.

Neutralizing antibodies, which bind to viral glycoproteins and prevent viral entry, have been considered a key HCMV vaccine development efficacy endpoint [46,47,48]. We measured plasma HCMV neutralization activity against AD169r virus in human fibroblast and epithelial cell lines and against Toledo virus in fibroblast cells. Similarly to previously reported in HIV-uninfected pregnant women, we observed higher plasma-neutralizing antibody titers in cCMV-transmitting maternal and infant plasma compared to the non-transmitting group against these strains (Figure 3A). There were no statistically significant differences in ADCC-mediating antibodies, measured by the frequency of degranulated CD107a+ NK cells [49,50,51] (Figure 3B) or in ADCP-mediating Abs against AD169r or Toledo virus strains in cCMV-transmitting maternal plasma compared to that of the non-transmitting group (Figure 3C).

### 3.6. HCMV-Specific IgG Fc Binding Profiles in cCMV-Transmitting and Non-Transmitting WLWH

**Figure 4:**
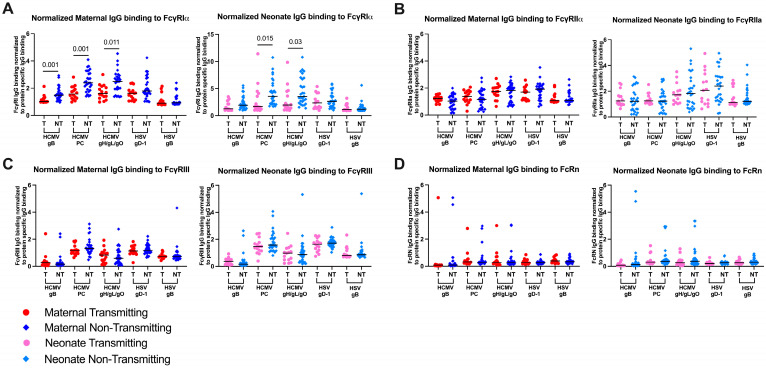
Higher normalized HCMV-specific IgG binding to FcγRIα in non-transmitting compared to transmitting samples. HCMV and HSV viral antigen IgG binding was measured using a modified Luminex assay using biotinylated Fc receptors and streptavidin-PE for detection and reported as MFI. Maternal (M) and neonate (NE) responses were measured within and between transmitting (T) and non-transmitting (NT) dyads. Responses were normalized to HCMV, HSV viral protein subunit specific IgG binding (Figure 2D). Normalized binding to (**A**) FcγRIα (CD64), (**B**) FcγRIIα (CD32), (**C**) FcγRIII (CD16) and (**D**) FcRN. (**A**–**D**) maternal (left) and neonate (right). Red circle indicates maternal transmitting group, pink circle indicates neonate transmitting group, dark blue diamond indicates maternal non-transmitting group and light blue diamond indicates neonate non-transmitting group. Horizontal black bars denote median. FDR corrected *p* values reported for comparisons within maternal or neonate samples for Wilcoxon’s signed-rank test (**A**–**D**) with *p* < 0.05 noted numerically.

To determine whether distinct Fc receptor engagement of HCMV-specific IgG occurs in cases of cCMV transmission in this cohort, we measured plasma HCMV and, for comparison, HSV glycoprotein-specific IgG binding to FcγR1α, FcγR2α, FcγR3 and FcRn receptors. We observed higher-magnitude plasma HCMV glycoprotein-specific IgG binding to FcγR1α, FcγR2α, FcγR3 and FcRn receptors in cCMV-transmitting compared to non-transmitting maternal and neonate plasma, but no differences in HSV glycoprotein-specific IgG responses between the groups (Supplemental Figure S4A–D), indicating this difference is specific to IgG directed against HCMV. We next compared Fc receptor engagement across dyads with varying plasma levels of viral glycoprotein-specific IgG responses by normalizing the HCMV glycoprotein-specific Fc receptor binding levels to the corresponding viral protein IgG binding level. This approach allows for evaluation of FcγR binding independently of virion protein-specific IgG levels, which differed between transmission groups (Figure 2D). After normalization, we observed higher-magnitude FcγR1α binding by HCMV gB, PC and gB/gH/gO trimer glycoprotein-specific IgG in cCMV non-transmitting compared to cCMV-transmitting women (Figure 4A left). This result was consistent in the corresponding neonatal plasma (Figure 4A right). The enhanced HCMV glycoprotein-specific IgG FcγR1α binding in non-transmitting women was unique among the Fc receptors examined (FcγR2α, FcγR3 and FcRn receptors; Figure 4B–D).

### 3.7. HCMV Glycoprotein-Specific IgG Binding to FcγR1α in HIV Seropositive and Seronegative Cohorts

HCMV-specific IgG binding to FcγRs and Fc-mediated effector antibody functions like ADCP have been previously identified as potential correlates of protection against cCMV in a U.S. cohort of HCMV transmitting and (*n* = 41) and non-transmitting (*n* = 40) mother infant dyads, which lacked HIV co-infection [18]. Although this HIV seronegative cohort differs in demographics characteristics from our HIV/HCMV co-infection cohort, we compared the relative fold change in normalized HCMV gB, PC and gHgLgO glycoprotein-specific IgG binding to FcγR1α across cohorts to explore the generalizability of these results. We observed a similar fold increase in HCMV glycoprotein-specific IgG FcγR1α binding levels in non-transmitting compared to cCMV-transmitting women in both HIV/HCMV co-infected (gB median 1.39, range 0.73–2.27; PC median 1.37, range 0.79–2.91; gHgLgO median 1.50, range 0.64–4.41) and HIV-seronegative cohorts (gB median 1.17, range 0.48–2.31; PC median 1.32, range 0.05–2.83; gHgLgO median 1.45, range 0.16–2.29) (Supplemental Figure S5). The consistency across cohorts underscores the importance of enhanced normalized HCMV glycoprotein-specific IgG FcγR1α binding as an antibody feature potentially important in protection against cCMV transmission risk independent of HIV status.

### 3.8. Application of Logistic Regression Modeling to Identify Meaningful Predictors of cCMV Transmission in WLWH

In an exploratory analysis of this highly dimensional and collinear dataset, we applied logistic regression modeling to identify the most relevant features of the measured maternal plasma IgG responses associated with cCMV transmission. Of the 25 antibody measure variables with significant univariate association with cCMV transmission (*p* < 0.05; Supplemental Table S2), seven key variables were identified by four variable selection modeling procedures (automated variable logistic regression, LASSO, elastic net and random forest modeling; Supplemental Table S3) as potentially important in predicting cCMV transmission. A final logistic regression model, containing these seven non-severely collinear (Variance Inflation Factor < 10) features was generated using a training subset, *n* = 13, of the overall cohort using 5-fold cross validation (Supplemental Table S4). Model performance was evaluated on the withheld testing set. Given the exploratory nature of this analysis in this cohort, we hypothesized that the following maternal plasma antibody measures may be the most meaningful in predicting risk and protection from cCMV transmission in WLWH, which will be important to assess in other HCMV seropositive transmission cohorts: (1) IgG binding to HCMV gB glycoprotein, (2) HCMV gB-specific IgG binding to FcγR3 receptor (3) Normalized HCMV PC-specific IgG binding to FcγR1α receptor, (4) HCMV gB-specific IgG binding to the neonatal receptor FcRn, (5) Neutralizing antibody titer against AD169r in fibroblast cells, (6) HCMV gB-specific IgG binding to FcγR1α receptor and (7) IgG binding to HCMV UL141 viral protein. No variable was retained as a statistically significant predictor of cCMV transmission (Supplemental Table S4).

## 4. Discussion

Investigating the antibody features and functions that are predictive of cCMV risk is crucial to reducing the impact of this extremely common cause of birth defects and brain damage across global populations. This work is particularly important to study in WLWH and HCMV who experience a high incidence of cCMV transmission. Our study leveraged biobanked samples from three historical cohorts of pregnant women living with HIV/HCMV co-infection to explore the immune correlates of cCMV transmission. We characterized the plasma antibody responses of 16 cCMV-transmitting compared to propensity-matched non-transmitting mother–neonate dyads (*n* = 29). We observed a trend towards higher-magnitude plasma HCMV-specific IgG responses, including HCMV glycoprotein-specific IgG concentrations, and higher neutralizing antibody titers in cCMV-transmitting compared to non-transmitting maternal-neonate dyads which may reflect more HCMV reactivation (magnitude or persistence of viremia). These results suggest that a high quantity of plasma HCMV-specific and neutralizing antibodies is not independently protective against cCMV acquisition but instead may be indicative of transmission risk. This association of higher HCMV-specific IgG responses in cCMV-transmitting compared to non-transmitting women is in line with observations in other cohorts [25]. Further, the observation of high neutralizing antibody responses being associated with vertical virus transmission risk is also in line with findings in studies of vertical HIV transmission [52,53,54]. Thus, a vaccine strategy focusing solely on eliciting high levels of HCMV-specific IgG binding and neutralizing antibodies may not be sufficient to prevent cCMV transmission. Yet, the distinct antibody profiles in cCMV transmitting women may represent an opportunity to predict transmission risk in the setting of maternal HCMV infection, as indicated in other cohorts [18,19,25,55,56].

To determine whether the observed increased HCMV-specific IgG responses in cCMV-transmitting women was attributable to timing of maternal HCMV infection or exposure, we explored whether there were differences in plasma HCMV-specific IgM detection, IgG avidity, or HCMV DNAemia between the cCMV-transmitting and non-transmitting women. Comparable frequency of HCMV-specific IgM and levels of high HCMV-specific IgG avidity alongside no detectable HCMV DNAemia in cCMV-transmitting and non-transmitting maternal groups supports the expected epidemiology that the majority women in this cohort were chronically infected with both HIV and HCMV. However, how these serologic markers relate to HCMV re-infection or re-activation is not well established.

Mounting evidence suggest that the key anti-viral non-neutralizing functional antibody responses of ADCP and ADCC may be associated with protection against cCMV transmission [18,19,57]. In this cohort of WLWH, plasma HCMV-specific ADCP responses were not significantly different between cCMV-transmitting and non-transmitting groups. The use of a monocyte cell line (THP-1) for this assessment, as opposed to primary monocytes, may be limiting the observed impact. Due to limited sample, the role of complement and opsonization in mediating ADCP was not assessed.

Interestingly, normalized HCMV-specific IgG FcγR1α, which is associated with ADCP, binding emerged as the primary antibody feature uniquely higher in non-transmitting compared to cCMV-transmitting WLWH. As noted above, we did not detect significant differences in ADCP, but this may reflect limitations of using a cell line (THP-1) for this assessment, as opposed to primary monocytes. In addition, due to limited sample, the role of complement and opsonization in mediating ADCP was not assessed. However, the observation that IgG FcγR1α binding may be protective is consistent with findings in an HIV-uninfected cohort [18] and suggests the potential for Fc-mediated effector functions and/or selective transplacental transfer of antibodies with specific Fc functions as a contributor to cCMV transmission risk, as reported for other placentally transferred virus-specific antibodies [55,58,59,60]. However, in this cohort of WLWH we observed comparable HCMV-specific FcγR3α binding, which is associated with ADCC, and similar HCMV ADCC responses. ADCC was recently identified as potentially associated with protection in cohorts of cCMV-transmitting and non-transmitting women without maternal HIV co-infection, underscoring the variation in potential immune correlates of placental virus transmission in distinct maternal demographics, co-infection and HCMV exposure status. These results provide further evidence of the unique immune landscape governing HIV and HCMV co-infection and its impact on cCMV transmission.

We applied logistic regression modeling in an attempt to identify the most relevant features of the measured maternal plasma IgG responses associated with cCMV transmission. We identified 7 variables as potential correlates of risk or protection against cCMV transmission in WLWH and HCMV but none were retained as statistically significant in the final model (Supplemental Table S4). Future work should use larger independent HCMV seropositive cohorts with and without HIV co-infection to identify the best performing predictive model of these 7 identified parameters that may predict cCMV transmission risk in chronically HCMV-infected populations.

Limitations of this study include the small sample size, retrospective study design with unknown maternal HCMV (primary, secondary, re-infection) infection status and unknown timing of neonatal infection and clinical outcomes. Additionally, due to limited sample availability, neonate cCMV screening was based primarily on plasma qPCR instead of the standard method utilizing saliva or urine [61]. HCMV DNAemia has considerable limitations in the diagnosis of acute HCMV infection, as viremia is short lived in comparison to viral shedding in urine or saliva, which can be detected for many months after primary infection [23]. Additionally, the frequency of HCMV viral detection or level difference in transmission groups may be more likely to exist in mucosal compartments where the virus is shed, such as urine or saliva, yet these samples were not collected in this cohort [56]. Lastly, although we focus only on plasma antibody responses, the role of the cellular responses, including T and NK cell function, as well as mucosal antibody responses, remain a critical gap for future analyses that collect relevant samples.

The elevated levels of neutralizing antibodies observed in cCMV-transmitting dyads may reflect recent HCMV reactivation. However, this finding has important implications for CMV vaccine development and our understanding of protective immunity against cCMV. This observation suggests that vaccines designed primarily to induce neutralizing antibodies may not be sufficient to prevent cCMV transmission, particularly in the setting of prior immunity. Instead, our findings support the development of vaccines that elicit a broader spectrum of antibody functions, including those that enhance Fc-mediated effector functions.

Future studies should prioritize identifying the epitopes of FcγR-activating antibodies that contribute to robust functional antiviral responses. As an example, ADCC- and ADCP-targeted antigens may differ from those involved in neutralization. Additionally, further characterization of protective HCMV-specific IgG Fc features, particularly their engagement with FcγR1α receptors, and a better understanding of how these interactions mediate antiviral functions may provide guidance to the development of vaccines that are effective in preventing cCMV transmission across diverse global populations. The lack of an independent association between maternal plasma-neutralizing antibody titers and reduced cCMV risk highlights the importance of considering alternative immunologic targets for HCMV vaccine efficacy. Eliciting antibodies with functional properties, such as FcγR1α engagement to promote ADCP, will be an important factor to evaluate in future HCMV vaccine candidates. This work is especially significant for solidifying immune correlates of protection in HCMV seropositive populations, where the majority of cCMV transmission occurs across the globe, as we move closer to a licensed vaccine against HCMV.

## Figures and Tables

**Table 1:** HCMV transmitting and non-transmitting dyads were matched by propensity score at a 1:2 ratio based on maternal age (>25 or <25), race (Black, White, Asian, not specified), ethnicity (Hispanic, non-Hispanic or not specified), gravida (number or births), parity (nulliparous or non-nulliparous), CD4+ T cell counts (>200 or <200 cells/mm^3^) and gestational age. HCMV viral copies are shown as viral copies/mL. The lower limit of detection is 250 copies/mL. “Not specified” indicates that the information was not available in cohort databases. ND indicates not detected. *p* values reported for Wilcoxon’s signed-rank test; Pearson’s Chi-squared test; Fisher’s exact test.

Baseline Characteristics of Transmitting and Non-Transmitting Mother-Neonate Dyads			
Mother-Neonate Dyad Characteristics	HCMV Transmitting	HCMV Non-Transmitting	*p*-Value
	(n = 16)	(n = 29)	
**Maternal age, yr, median (range)**	26.5 (16–43)	26 (19–41)	0.8
>25, n (%)	9 (56.3%)	15 (51.7%)	
≤25, n (%)	7 (43.8%)	14 (48.3%)	
**Maternal race, n (%)**			0.9
Black or African American	14 (87.5%)	22 (75.9%)	
White	1 (6.3%)	2 (6.9%)	
Asian	0 (0%)	0 (0%)	
Not specified	1 (6.3%)	5 (17.2%)	
**Maternal ethnicity, n (%)**			0.15
Hispanic	0 (0%)	5 (17.2%)	
Non-hispanic	15 (93.8%)	24 (82.8%)	
Not specified	1 (6.3%)	0 (%)	
**Gravida, median (range)**	4 (1–7)	4(1–6)	0.9
**Parity, n (%)**			0.9
nulliparous	12 (75.0%)	21 (72.4%)	
non-nulliparous	2 (12.5%)	4 (13.8%)	
Not specified	2 (12.5%)	4 (13.8%)	
**CD4+ T cell count, median (range)**	424.3 (19.5–1263.5)	440.5 (98.5–1026.5)	>0.9
>200, n (%)	12 (75.0%)	24 (82.8%)	
≤200, n (%)	4 (25.0%)	5 (17.2%)	
**HIV plasma viral load, median (range)**	404.3 (50–41,500)	400 (20–89,928)	0.4
**Maternal plasma HCMV DNAemia, n (%)**			
Positive	0 (0%)	0 (0%)	
Negative	16 (100%)	29 (100%)	
**Maternal plasma HCMV viral copies, median (range)**	ND	ND	
**Neonate plasma HCMV viral copies, median (range)**	250–2469	ND	
**Maternal HCMV IgM seropositivity n (%)**	1 (6.3%)	0 (0%)	0.4
**Cohort, n (%)**			
PACTG (study 316)	12 (26.7%)	21 (46.7%)	
IMPAACT (study P1025)	2 (4.4%)	4 (8.9%)	
NISDI (Perinatal protocol)	2 (4.4%)	4 (8.9%)	

## Data Availability

Data are available upon request to the corresponding author (sap4017@med.cornell.edu).

## Supplementary Materials

The following supporting information can be downloaded at: https://www.mdpi.com/article/10.3390/v17030325/s1, Table S1: Baseline characteristics of NISDI Perinatal protocol neonates; Figure S1: Maternal HIV and D4+ T cell levels; Figure S2: Impaired transplacental IgG transfer in transmitting and non-transmitting dyads; Figure S3: ADCC gating schema; Figure S4: Higher-magnitude HCMV-specific IgG binding to FcγRIα, FcγRIIα, FcγRIII and FcRn in samples from transmitting and non-transmitting maternal and neonate sera; Table S2: The 25 variables with univariate association with HCMV transmission; Table S3: Emergence of 7 main variables of importance via variable selection modeling procedures; Table S4: Logistic Regression data-driven model with 7 meaningful variables does not yield significant predictors of cCMV transmission; Figure S5: Normalized FcγR1α in HIV seropositive and HIV seronegative cohorts.

## Abbreviations

The following abbreviations are used in this manuscript:

HCMV	Human cytomegalovirus
cCMV	Congenital cytomegalovirus
HIV	Human immunodeficiency virus
WLWH	Women living with HIV
ADCP	Antibody-dependent cellular phagocytosis
ADCC	Antibody-dependent cellular cytotoxicity
ART	Antiretroviral therapy
NISDI	NICHD International Site Development Initiative
PACTG	Pediatric AIDS Clinical Trials Group
IMPAACT	International Maternal Pediatric Adolescent AIDS Clinical Trials Group
PBMC	Peripheral blood mononuclear cells
qPCR	Quantitative polymerase chain reaction
PTID	Patient ID
