# Supplementary material for: Influence of Distinct Maternal Cytomegalovirus-Specific Neutralizing and Fc Receptor-Binding Responses on Congenital Cytomegalovirus Transmission in HIV-Exposed Neonates
Source: Viruses. 2025 Feb 26;17(3):325. doi: 10.3390/v17030325 (PMC11946089; doi:10.3390/v17030325)
Supplement: Supplementary file 1 [file viruses-17-00325-s001.zip › viruses-3466932-supplementary.pdf]

Supplemental Table S1. Identification of 2 cases of cCMV by HCMV immediate early (IE-1) gene-based qPCR screen of 396 neonate plasma from the NICHD International Site Development Initiative (NISDI, Perinatal protocol). “Not specified” indicates that the information was not available in cohort databases.

**Supplemental Table 1: Baseline characteristics of NISDI Perinatal protocol neonates**	
**Neonate characteristics**	**(n=396)**
**Sapmling timeoint, days, median (range)**	3.14 (0-64)
>14 days, n (%)	392 (98.99%)
≤14 days, n (%)	4 (1.01%)
**Neonate race, n (%)**	
Black or African American	60 (15.2%)
White	209 (52.8%)
Multi Race	7 (1.8%)
Not specified	120 (30.3%)
**Neonate ethnicity, n (%)**	
Hispanic	109 (27.5%)
Non-hispanic	269 (67.9%)
Not specified	18 (4.5%)
**Neonate sex, n (%)**	
Female	194 (48.9%)
Male	199 (50.3%)
Not specified	3 (0.8%)
**HCMV transmission status, n (%)**	
Negative (<250 copies/mL)	394 (99.5%)
Positive (>250 copies/mL)	2 (0.5%)
**HCMV viral load for cCMV Positive cases (copies/mL)**	
PTID 70017A	2469.9
PTID 72074A	392.6

Supplemental Figure S1. Maternal HIV and CD4+ T cell levels.

Clinical features associated with HIV of maternal samples in transmitting (T) and non-transmitting (NT) maternal samples. (**A**) Plasma HIV viral load **(B**) CD4+ T cell count. Red circle indicates maternal transmitting and dark blue diamond indicates maternal non-transmitting samples. Horizontal black bars denote mean. P values reported for Wilcoxon's signed-rank test (A-B) with $P < 0.05$ noted numerically.

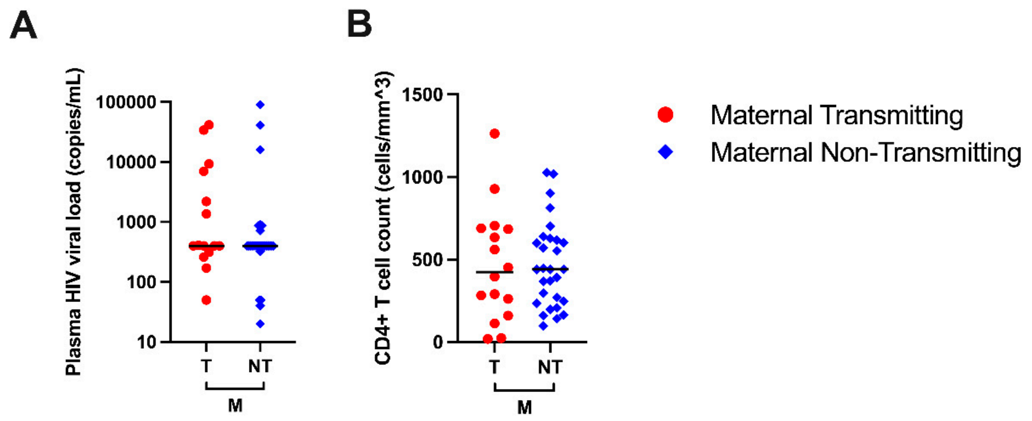

Supplemental Figure S2. Impaired transplacental IgG transfer in transmitting and non-transmitting dyads.

Maternal to neonate transplacental IgG transfer ratios between transmitting (T) and non-transmitting (NT) dyads. Total and HCMV specific antibody levels were measured by ELISA, viral protein subunit specific IgG binding was measured with a Luminex based assay at the specified plasma dilution. High avidity HCMV was measured by a modified ELISA. Transfer ratio in each paired maternal –neonate was calculated as neonate/maternal value. (**A**) Total IgG transfer ratios (**B**) HCMV virion IgG transfer ratios against AD169r and Toledo HCMV strains. (C) HCMV, HSV, and HIV glycoprotein specific IgG transfer ratios (**D**) High avidity virion IgG transfer ratios

Red circle indicates transmitting maternal-neonate dyad, dark blue diamond indicates non-transmitting maternal-neonate dyad. Horizontal black bars denote median. FDR corrected P values for comparisons between maternal samples reported for Wilcoxon's signed-rank test (A-D) with P < 0.05 noted numerically.

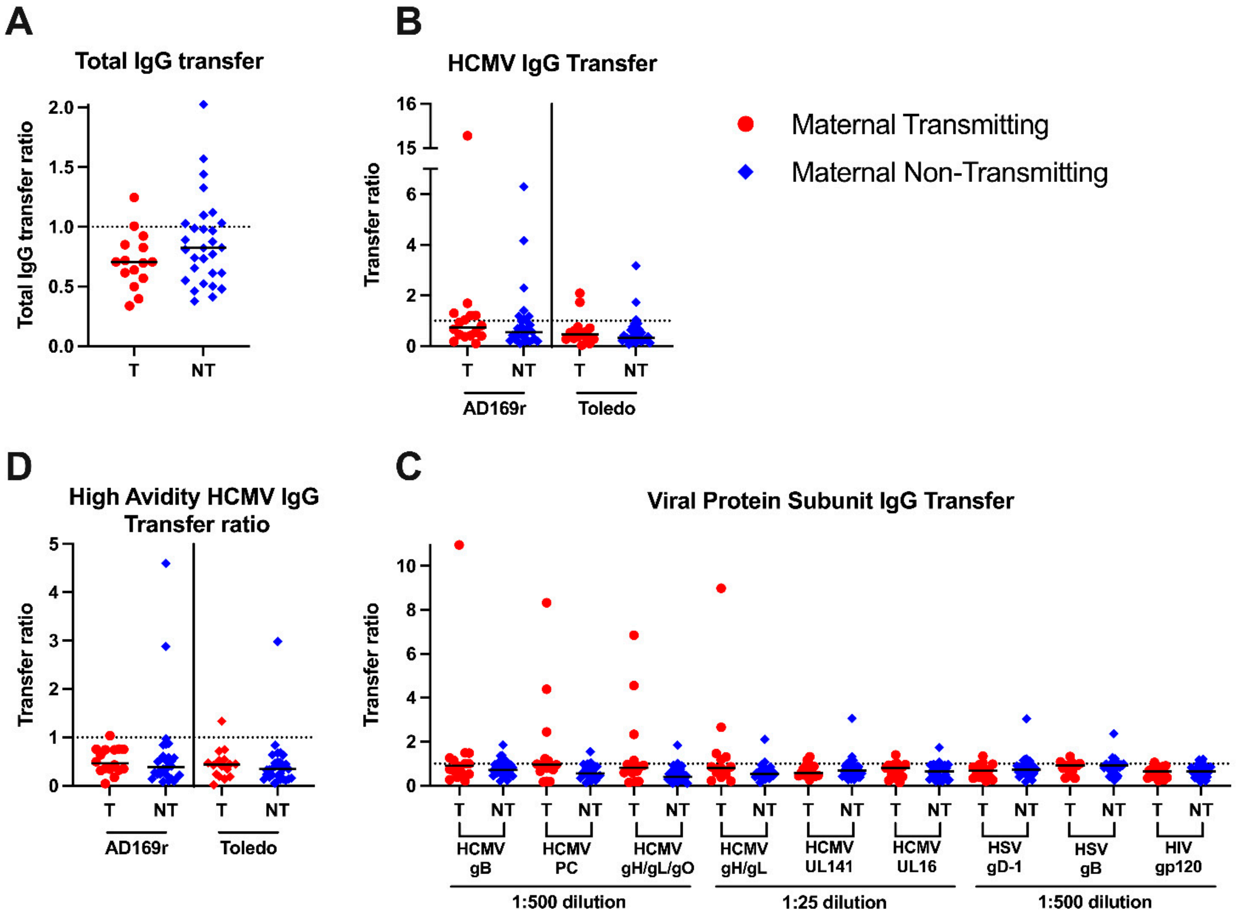

Supplemental Figure S3. ADCC gating schema

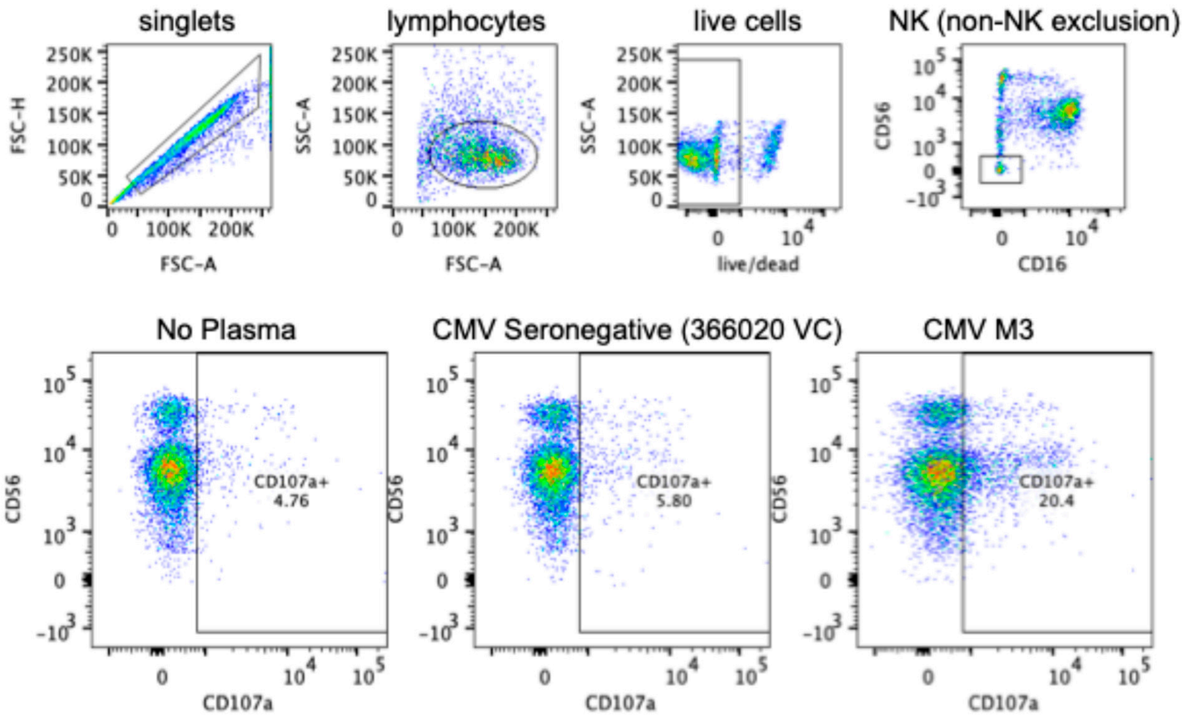

Supplemental Figure S4. Higher magnitude HCMV-specific IgG binding to FcγRIα, FcγRIIα, FcγRIII, and FcRn in samples from transmitting and non-transmitting maternal and neonate sera.

HCMV and HSV viral antigen IgG binding was measured using a modified Luminex assay using biotinylated Fc receptors and streptavidin-PE for detection and reported as MFI. Maternal (M) and neonate (NE) responses were measured within and between transmitting (T) and non-transmitting (NT) dyads. (**A**) FcγRIα (CD64), (**B**) FcγRIIα (CD32), (**C**) FcγRIII (CD16), and (**D**) FcRn. (**A-D**) maternal (left) and neonate (right).

Red circle indicates maternal transmitting group, pink circle indicates neonate transmitting group, dark blue diamond indicates maternal non-transmitting group, and light blue diamond indicates neonate non-transmitting group. Horizontal black bars denote median. FDR corrected P values for comparisons within maternal or neonate samples reported for Wilcoxon's signed-rank test (A-D) with P < 0.05 noted numerically.

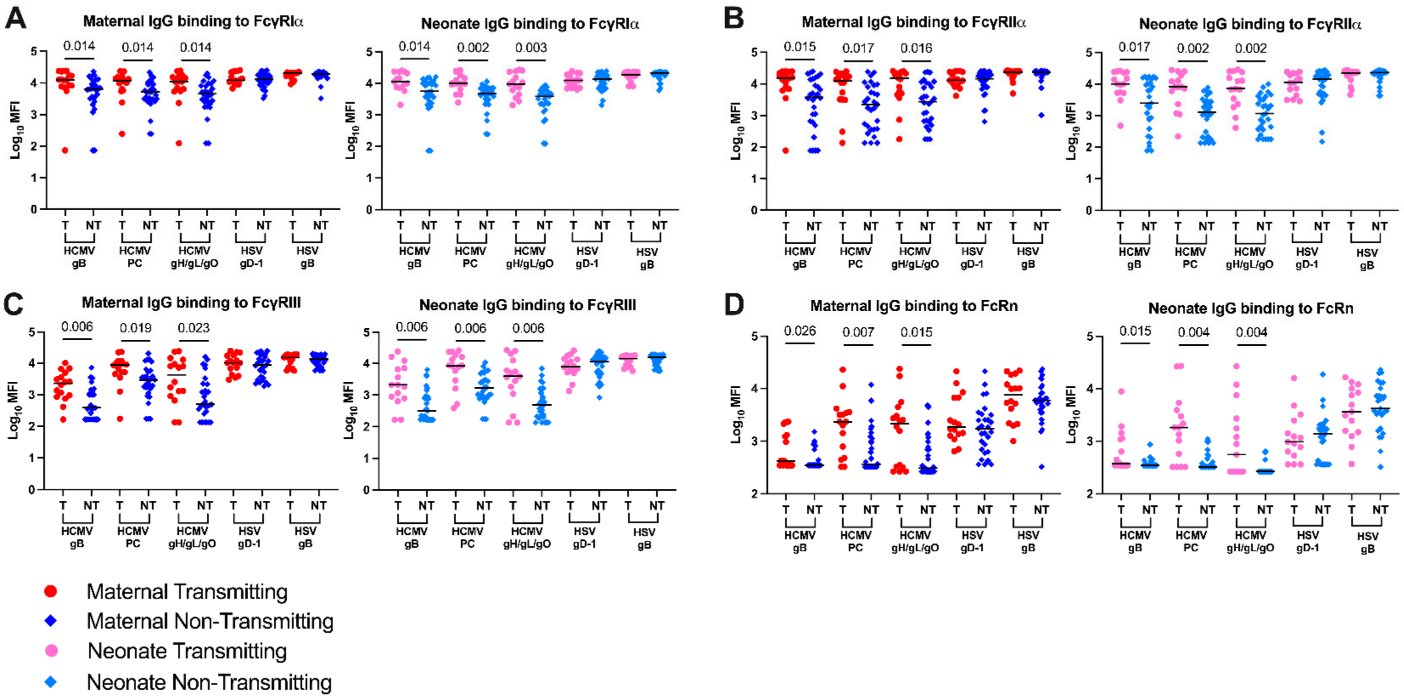

Supplemental Table S2. Twenty-five variables were selected from an initial pool of 86 variables based on a significance level of an FDR corrected p-value less than 0.05. "Measurement' refers to the variable name, while "Assay Measurement' denotes the assay from which the variable is quantified, "Normalized" indicated whether the variable is derived from more than one measurement. 'Directionality' specifies whether the median value of the variable is significantly higher or lower in Non-transmitting (NT) maternal compared to Transmitting (T) plasma.

Measurement	Assay Measurement	Normalized	FDR corrected p-value	Directionality
Total IgG concentration	ELISA	No	0.067	Lower in NT
HCMV gB binding	BAMA	No	0.009	Lower in NT
HCMV PC binding	BAMA	No	0.009	Lower in NT
HCMV gHgLgO binding	BAMA	No	0.009	Lower in NT
HCMV gHgL binding	BAMA	No	0.016	Lower in NT
HCMV UL141 binding	BAMA	No	0.004	Lower in NT
FcγR1α HCMV gB binding	FcγR1 BAMA	No	0.014	Lower in NT
FcγR1α HCMV PC binding	FcγR1 BAMA	No	0.002	Lower in NT
FcγR1α HCMV gHgLgO binding	FcγR1 BAMA	No	0.003	Lower in NT
FcγR2α HCMV gB binding	FcγR2 BAMA	No	0.015	Lower in NT
FcγR2α HCMV PC binding	FcγR2 BAMA	No	0.017	Lower in NT
FcγR2α HCMV gHgLgO binding	FcγR2 BAMA	No	0.016	Lower in NT
FcγR3 HCMV gB binding	FcγR3 BAMA	No	0.006	Lower in NT
FcγR3 HCMV PC binding	FcγR3 BAMA	No	0.019	Lower in NT
FcγR3 HCMV gHgLgO binding	FcγR3 BAMA	No	0.023	Lower in NT
FcRn HCMV gB binding	FcRn BAMA	No	0.026	Lower in NT
FcRn HCMV PC binding	FcRn BAMA	No	0.007	Lower in NT
FcRn HCMV gHgLgO binding	FcRn BAMA	No	0.015	Lower in NT
Normalized FcγR1α HCMV gB binding	FcγR1 BAMA	Yes	0.001	Higher in NT
Normalized FcγR1α HCMV PC binding	FcγR1 BAMA	Yes	0.001	Higher in NT
Normalized FcγR1α HCMV gHgLgO binding	FcγR1 BAMA	Yes	0.011	Higher in NT
Neutralization of AD169r HCMV with epithelial cells	Neutralization	No	0.003	Lower in NT
Neutralization of AD169r HCMV with fibroblast cells	Neutralization	No	0.006	Lower in NT
Neutralization of Toledo HCMV with fibroblast cells	Neutralization	No	0.025	Lower in NT
ADCP of Toledo HCMV	ADCP	No	0.024	Lower in NT

Supplemental Table S3. Seven variables were selected as 'important' from the 25 variables with FDR corrected p-value less than 0.05. "Measurement' refers to the variable name, while "Assay Measurement' denotes the assay from which the variable is quantified, "Normalized" indicated whether the variable is derived from more than one measurement. Four variable selection modeling procedures were employed: LASSO, Stepwise automated variable selection logistic regression, Elastic net, and Random forest modeling. Importance was determined for Random Forest as a value > 1. '# of models" indicates the number of models in which the measurement was classified as important. As a measurement of collinearity, variance inflation factor (VIF) is noted.

Measurement	Assay Measurement	Normalized	Lasso	Stepwise	Elastic net	Random forest	Importance cut-off	# of Models	VIF
HCMV gB binding	BAMA	No	Not Important	Important	Not Important	Important	>1	2	5.419
FcγR3 HCMV gB binding	FcγR3 BAMA	No	Important	Important	Important	Important	>1	4	2.907
Normalized FcγR1α HCMV PC binding	FcγR1 BAMA	Yes	Important	Not Important	Important	Important	>1	3	5.011
FcRn HCMV gHgLgO binding	FcRn BAMA	No	Not Important	Important	Not Important	Important	>1	2	3.441
Neutralization of AD169r HCMV with fibroblast cells	Neutralization	No	Important	Important	Not Important	Important	>1	3	1.338
FcγR1α HCMV gB binding	FcγR1 BAMA	No	Not Important	Important	Not Important	Important	>1	2	2.362
HCMV UL141 binding	BAMA	No	Important	Not Important	Not Important	Important	>1	2	3.149

Supplemental Table S4. Data-driven model, containing non-collinear features, was generated on a training subset of the overall cohort using 5-fold cross validation. “Measurement’ refers to the variable name, while “Assay Measurement’ denotes the assay from which the variable is quantified, “Normalized” indicated whether the variable is derived from more than one measurement. Adjusted Odds ratio noted, with OR>1 indicating an increasing value of the measurement (predictor) is associated with an increased odds of HCMV transmission. Confidence interval and p-value as noted.

Measurement	Assay Measurement	Normalized	Adjusted Odds Ratio	Lower 95% CI	Upper 95% CI	p-value
HCMV gB binding	BAMA	No	0.036	0	0.936	0.069
FcγR3 HCMV gB binding	FcγR3 BAMA	No	13.724	0.431	1075.704	0.17
Normalized FcγR1α HCMV PC binding	FcγR1 BAMA	Yes	0.04	0	3.684	0.204
FcRn HCMV gHgLgO binding	FcRn BAMA	No	0.15	0.003	4.061	0.287
Neutralization of AD169r HCMV with fibroblast cells	Neutralization	No	2.306	0.214	35.1	0.489
FcγR1α HCMV gB binding	FcγR1 BAMA	No	1.714	0.004	468.925	0.848
HCMV UL141 binding	BAMA	No	55.03	1.469	30144.805	0.88

Supplemental Table S5. Raw and FDR corrected P values for comparisons within maternal or neonate samples reported for Wilcoxon's signed-rank test in Table 1, Figures 1-4, Supplemental Figures 1,2,4. Values noted per figure for each comparison within maternal or neonate samples as noted in the comparison column. M-T (red letters) indicates maternal transmitting group, NE-T (pink letters) indicates neonate transmitting group, M-NT (dark blue letters) indicates maternal non-transmitting group, NE-NT (light blue letters) indicates neonate non-transmitting group. Test indicates the statistical method used for each comparison.

Supplemental Table 5

Figure	Measurement	Comparison	Test	p-value	FDR corrected p-value
Demographics	Gestational age	M-T to M-NT	Wilcoxon rank sum	0.071	
	Maternal age	M-T to M-NT	Fisher's exact test	0.8	
	Maternal race	M-T to M-NT	Person's Chi-squared test	> 0.9	
	Maternal ethnicity	M-T to M-NT	Person's Chi-squared test	0.15	
	Maternal gravida	M-T to M-NT	Wilcoxon rank sum	0.9	
	Maternal parity	M-T to M-NT	Person's Chi-squared test	> 0.9	
Supplemental Figure 1	Maternal plasma HIV viral load	M-T to M-NT	Wilcoxon rank sum	0.4	0.6
	Maternal CD4+ T cell count	M-T to M-NT	Wilcoxon rank sum	>0.9	>0.9
Figure 1A	Total Serum IgG	M-T to M-NT	Wilcoxon rank sum	0.022	0.067
		NE-T to NE-NT	Wilcoxon rank sum	0.3	0.3
Figure 1B	HCMV IgG Binding (AD169r)	M-T to M-NT	Wilcoxon rank sum	0.6	0.6
		NE-T to NE-NT	Wilcoxon rank sum	0.077	0.3
	HCMV IgG Binding (Toledo)	M-T to M-NT	Wilcoxon rank sum	0.5	0.6
		NE-T to NE-NT	Wilcoxon rank sum	0.2	0.5
Figure 1C	HCMV IgG1 binding	M-T to M-NT	Wilcoxon rank sum	0.5	>0.9
		NE-T to NE-NT	Wilcoxon rank sum	>0.9	>0.9
	HCMV IgG3 binding	M-T to M-NT	Wilcoxon rank sum	0.2	0.5
		NE-T to NE-NT	Wilcoxon rank sum	0.7	0.7
Figure 1D	HCMV gB binding	M-T to M-NT	Wilcoxon rank sum	0.003	0.009
	HCMV PC binding	M-T to M-NT	Wilcoxon rank sum	0.004	0.009
	HCMV gHgLgO binding	M-T to M-NT	Wilcoxon rank sum	0.004	0.009
	HCMV gHgL binding	M-T to M-NT	Wilcoxon rank sum	0.009	0.016
	HCMV UL141 bidning	M-T to M-NT	Wilcoxon rank sum	<0.001	0.004
	HCMV UL16 binding	M-T to M-NT	Wilcoxon rank sum	0.3	0.4
	HSV gD-1 binding	M-T to M-NT	Wilcoxon rank sum	0.4	0.6
	HSV gB binding	M-T to M-NT	Wilcoxon rank sum	0.5	0.6
	HIV gp120 binding	M-T to M-NT	Wilcoxon rank sum	0.5	0.6
	HCMV gB binding	NE-T to NE-NT	Wilcoxon rank sum	0.005	0.011
	HCMV PC binding	NE-T to NE-NT	Wilcoxon rank sum	<0.001	0.004
	HCMV gHgLgO binding	NE-T to NE-NT	Wilcoxon rank sum	<0.001	0.004
	HCMV gHgL binding	NE-T to NE-NT	Wilcoxon rank sum	0.003	0.009
	HCMV UL141 bidning	NE-T to NE-NT	Wilcoxon rank sum	0.003	0.009
	HCMV UL16 binding	NE-T to NE-NT	Wilcoxon rank sum	0.6	0.7
	HSV gD-1 binding	NE-T to NE-NT	Wilcoxon rank sum	>0.9	>0.9
	HSV gB binding	NE-T to NE-NT	Wilcoxon rank sum	>0.9	>0.9
	HIV gp120 binding	NE-T to NE-NT	Wilcoxon rank sum	0.6	0.7
Figure 2A	HCMV IgM	M-T to M-NT	Fisher's exact test	0.4	0.6
Figure 2B	High Avidity IgG (AD169r)	M-T to M-NT	Wilcoxon rank sum	0.5	0.5
		NE-T to NE-NT	Wilcoxon rank sum	0.95	0.3

		M-T to NE-T	Wilcoxon rank sum	0.001	0.002
	High Avidity IgG (Toledo)	M-T to M-NT	Wilcoxon rank sum	0.3	0.4
		NE-T to NE-NT	Wilcoxon rank sum	0.2	0.3
Figure 2C	HCMV viral load	M-T to M-NT	Wilcoxon rank sum	All below limit of detection	
Figure 2D	High avidity HCMV gB binding	M-T to M-NT	Wilcoxon rank sum	>0.9	>0.9
	High avidity HCMV PC binding	M-T to M-NT	Wilcoxon rank sum	0.8	>0.9
	High avidity HCMV gHgLgO binding	M-T to M-NT	Wilcoxon rank sum	0.6	>0.9
	High avidity HCMV gHgL binding	M-T to M-NT	Wilcoxon rank sum	0.7	>0.9
	High avidity HCMV UL141 bidning	M-T to M-NT	Wilcoxon rank sum	0.2	0.8
	High avidity HCMV UL16 binding	M-T to M-NT	Wilcoxon rank sum	0.8	>0.9
	High avidity HSV gD-1 binding	M-T to M-NT	Wilcoxon rank sum	0.12	0.8
	High avidity HSV gB binding	M-T to M-NT	Wilcoxon rank sum	0.9	>0.9
	High avidity HCMV gB binding	NE-T to NE-NT	Wilcoxon rank sum	0.9	>0.9
	High avidity HCMV PC binding	NE-T to NE-NT	Wilcoxon rank sum	0.4	>0.9
	High avidity HCMV gHgLgO binding	NE-T to NE-NT	Wilcoxon rank sum	0.7	>0.9
	High avidity HCMV gHgL binding	NE-T to NE-NT	Wilcoxon rank sum	0.2	0.8
	High avidity HCMV UL141 bidning	NE-T to NE-NT	Wilcoxon rank sum	0.075	0.8
	High avidity HCMV UL16 binding	NE-T to NE-NT	Wilcoxon rank sum	0.7	>0.9
	High avidity HSV gD-1 binding	NE-T to NE-NT	Wilcoxon rank sum	0.4	>0.9
	High avidity HSV gB binding	NE-T to NE-NT	Wilcoxon rank sum	0.6	>0.9
Supplemental Figure 2A	Total IgG transfer	M-T to M-NT	Wilcoxon rank sum	0.2	0.3
Supplemental Figure 2B	HCMV IgG transfer (AD169r)	M-T to M-NT	Wilcoxon rank sum	0.4	NS
	HCMV IgG transfer (Toledo)	M-T to M-NT	Wilcoxon rank sum	0.4	NS
Supplemental Figure 2C	Subunit IgG transfer HCMV gB	M-T to M-NT	Wilcoxon rank sum	0.4	0.8
	Subunit IgG transfer HCMV PC	M-T to M-NT	Wilcoxon rank sum	0.056	0.3
	Subunit IgG transfer HCMV gH/gL/gO	M-T to M-NT	Wilcoxon rank sum	0.034	0.3
	Subunit IgG transfer HCMV gH/gL	M-T to M-NT	Wilcoxon rank sum	0.088	0.3
	Subunit IgG transfer HCMV UL141	M-T to M-NT	Wilcoxon rank sum	0.7	0.8
	Subunit IgG transfer HCMV UL16	M-T to M-NT	Wilcoxon rank sum	>0.9	>0.9
	Subunit IgG transfer HSV gD-1	M-T to M-NT	Wilcoxon rank sum	0.4	0.8
	Subunit IgG transfer HSV gB	M-T to M-NT	Wilcoxon rank sum	0.7	0.8
	Subunit IgG transfer HIV gp120	M-T to M-NT	Wilcoxon rank sum	0.6	0.8
Supplemental Figure 2D	High avidity HCMV IgG transfer (AD169r)	M-T to M-NT	Wilcoxon rank sum	0.4	NS
	High avidity HCMV IgG transfer (Toledo)	M-T to M-NT	Wilcoxon rank sum	0.4	NS
Figure 3A	HCMV neutralization (AD169r - Epithelial)	M-T to M-NT	Wilcoxon rank sum	<0.001	0.003
		NE-T to NE-NT	Wilcoxon rank sum	0.006	0.013
		M-T to NE-T	Wilcoxon rank sum	0.2	0.5
		M-NT to NE-NT	Wilcoxon rank sum	0.9	>0.9
	HCMV neutralization (AD169r - Fibroblast)	M-T to M-NT	Wilcoxon rank sum	0.002	0.006
		NE-T to NE-NT	Wilcoxon rank sum	0.032	0.035

		M-T to NE-T	Wilcoxon rank sum	0.3	0.5
		M-NT to NE-NT	Wilcoxon rank sum	>0.9	>0.9
	HCMV neutralization (Toledo - Fibroblast)	M-T to M-NT	Wilcoxon rank sum	0.017	0.025
		NE-T to NE-NT	Wilcoxon rank sum	0.035	0.035
		M-T to NE-T	Wilcoxon rank sum	0.5	0.5
		M-NT to NE-NT	Wilcoxon rank sum	0.2	0.7
Figure 3B	ADCC	M-T to M-NT	Wilcoxon rank sum	>0.9	>0.9
Figure 3C	ADCP (AD169r)	M-T to M-NT	Wilcoxon rank sum	0.13	0.3
		NE-T to NE-NT	Wilcoxon rank sum	0.2	0.4
		M-T to NE-T	Wilcoxon rank sum	0.3	0.3
		M-NT to NE-NT	Wilcoxon rank sum	0.5	0.5
	ADCP (Toledo)	M-T to M-NT	Wilcoxon rank sum	0.024	0.12
		M-T to NE-T	Wilcoxon rank sum	0.9	NS
		M-NT to NE-NT	Wilcoxon rank sum	0.6	NS
Supplemental Figure 4A	HCMV gB binding with FcγRI	M-T to M-NT	Wilcoxon rank sum	0.007	0.014
	HCMV PC binding FcγRI	M-T to M-NT	Wilcoxon rank sum	0.008	0.014
	HCMV gHgLgO binding FcγRI	M-T to M-NT	Wilcoxon rank sum	0.007	0.014
	HSV gD-1 binding FcγRI	M-T to M-NT	Wilcoxon rank sum	0.7	>0.9
	HSV gB binding FcγRI	M-T to M-NT	Wilcoxon rank sum	>0.9	>0.9
	HCMV gB binding with FcγRI	NE-T to NE-NT	Wilcoxon rank sum	0.009	0.014
	HCMV PC binding FcγRI	NE-T to NE-NT	Wilcoxon rank sum	<0.001	0.002
	HCMV gHgLgO binding FcγRI	NE-T to NE-NT	Wilcoxon rank sum	<0.001	0.003
	HSV gD-1 binding FcγRI	NE-T to NE-NT	Wilcoxon rank sum	0.7	>0.9
	HSV gB binding FcγRI	NE-T to NE-NT	Wilcoxon rank sum	>0.9	>0.9
Supplemental Figure 4B	HCMV gB binding with FcγRIIa	M-T to M-NT	Wilcoxon rank sum	0.005	0.015
	HCMV PC binding FcγRIIa	M-T to M-NT	Wilcoxon rank sum	0.01	0.017
	HCMV gHgLgO binding FcγRIIa	M-T to M-NT	Wilcoxon rank sum	0.006	0.016
	HSV gD-1 binding FcγRIIa	M-T to M-NT	Wilcoxon rank sum	0.7	0.8
	HSV gB binding FcγRIIa	M-T to M-NT	Wilcoxon rank sum	>0.9	>0.9
	HCMV gB binding FcγRIIa	NE-T to NE-NT	Wilcoxon rank sum	0.01	0.017
	HCMV PC binding FcγRIIa	NE-T to NE-NT	Wilcoxon rank sum	<0.001	0.002
	HCMV gHgLgO binding FcγRIIa	NE-T to NE-NT	Wilcoxon rank sum	<0.001	0.002
	HSV gD-1 binding FcγRIIa	NE-T to NE-NT	Wilcoxon rank sum	0.5	0.8
	HSV gB binding FcγRIIa	NE-T to NE-NT	Wilcoxon rank sum	0.7	0.8
Supplemental Figure 4C	HCMV gB binding with FcγRIIIa	M-T to M-NT	Wilcoxon rank sum	0.002	0.006
	HCMV PC binding FcγRIIIa	M-T to M-NT	Wilcoxon rank sum	0.01	0.019
	HCMV gHgLgO binding FcγRIIIa	M-T to M-NT	Wilcoxon rank sum	0.014	0.023
	HSV gD-1 binding FcγRIIIa	M-T to M-NT	Wilcoxon rank sum	0.5	0.6
	HSV gB binding FcγRIIIa	M-T to M-NT	Wilcoxon rank sum	0.9	0.9
	HCMV gB binding with FcγRIIIa	NE-T to NE-NT	Wilcoxon rank sum	0.002	0.006

	HCMV PC binding FcγRIIIa	NE-T to NE-NT	Wilcoxon rank sum	<0.001	0.006
	HCMV gHgLgO binding FcγRIIIa	NE-T to NE-NT	Wilcoxon rank sum	0.002	0.006
	HSV gD-1 binding FcγRIIIa	NE-T to NE-NT	Wilcoxon rank sum	0.9	0.9
	HSV gB binding FcγRIIIa	NE-T to NE-NT	Wilcoxon rank sum	0.5	0.6
Supplemental Figure 4D	HCMV gB binding with FcγRN	M-T to M-NT	Wilcoxon rank sum	0.015	0.026
	HCMV PC binding FcγRN	M-T to M-NT	Wilcoxon rank sum	0.002	0.007
	HCMV gHgLgO binding FcγRN	M-T to M-NT	Wilcoxon rank sum	0.008	0.015
	HSV gD-1 binding FcγRN	M-T to M-NT	Wilcoxon rank sum	0.3	0.4
	HSV gB binding FcγRN	M-T to M-NT	Wilcoxon rank sum	0.6	0.7
	HCMV gB binding with FcγRN	NE-T to NE-NT	Wilcoxon rank sum	0.006	0.015
	HCMV PC binding FcγRN	NE-T to NE-NT	Wilcoxon rank sum	<0.001	0.004
	HCMV gHgLgO binding FcγRN	NE-T to NE-NT	Wilcoxon rank sum	<0.001	0.004
	HSV gD-1 binding FcγRN	NE-T to NE-NT	Wilcoxon rank sum	0.7	0.7
	HSV gB binding FcγRN	NE-T to NE-NT	Wilcoxon rank sum	0.7	0.7
Figure 4A	Normalized HCMV gB binding with FcγRI	M-T to M-NT	Wilcoxon rank sum	<0.001	0.001
	Normalized HCMV PC binding FcγRI	M-T to M-NT	Wilcoxon rank sum	<0.001	0.001
	Normalized HCMV gHgLgO binding FcγRI	M-T to M-NT	Wilcoxon rank sum	0.003	0.011
	Normalized HSV gD-1 binding FcγRI	M-T to M-NT	Wilcoxon rank sum	0.11	0.2
	Normalized HSV gB binding FcγRI	M-T to M-NT	Wilcoxon rank sum	0.5	0.6
	Normalized HCMV gB binding with FcγRI	NE-T to NE-NT	Wilcoxon rank sum	0.074	0.12
	Normalized HCMV PC binding FcγRI	NE-T to NE-NT	Wilcoxon rank sum	0.006	0.015
	Normalized HCMV gHgLgO binding FcγRI	NE-T to NE-NT	Wilcoxon rank sum	0.015	0.03
	Normalized HSV gD-1 binding FcγRI	NE-T to NE-NT	Wilcoxon rank sum	0.5	0.6
	Normalized HSV gB binding FcγRI	NE-T to NE-NT	Wilcoxon rank sum	>0.9	>0.9
Figure 4B	Normalized HCMV gB binding with FcγRIIa	M-T to M-NT	Wilcoxon rank sum	0.2	0.8
	Normalized HCMV PC binding FcγRIIa	M-T to M-NT	Wilcoxon rank sum	0.4	0.8
	Normalized HCMV gHgLgO binding FcγRIIa	M-T to M-NT	Wilcoxon rank sum	0.7	0.8
	Normalized HSV gD-1 binding FcγRIIa	M-T to M-NT	Wilcoxon rank sum	0.3	0.8
	Normalized HSV gB binding FcγRIIa	M-T to M-NT	Wilcoxon rank sum	0.4	0.8
	Normalized HCMV gB binding FcγRIIa	NE-T to NE-NT	Wilcoxon rank sum	0.6	0.8
	Normalized HCMV PC binding FcγRIIa	NE-T to NE-NT	Wilcoxon rank sum	0.8	0.9
	normalized HCMV gHgLgO binding FcγRIIa	NE-T to NE-NT	Wilcoxon rank sum	>0.9	>0.9
	Normalized HSV gD-1 binding FcγRIIa	NE-T to NE-NT	Wilcoxon rank sum	0.5	0.8
	Normalized HSV gB binding FcγRIIa	NE-T to NE-NT	Wilcoxon rank sum	0.4	0.8
Figure 4C	Normalized HCMV gB binding with FcγRIIIa	M-T to M-NT	Wilcoxon rank sum	0.2	0.5
	Normalized HCMV PC binding FcγRIIIa	M-T to M-NT	Wilcoxon rank sum	0.11	0.5
	Normalized HCMV gHgLgO binding FcγRIIIa	M-T to M-NT	Wilcoxon rank sum	0.6	0.8
	Normalized HSV gD-1 binding FcγRIIIa	M-T to M-NT	Wilcoxon rank sum	0.5	0.8
	Normalized HSV gB binding FcγRIIIa	M-T to M-NT	Wilcoxon rank sum	0.9	>0.9

	Normalized HCMV gB binding with FcγRIIIa	NE-T to NE-NT	Wilcoxon rank sum	0.1	0.5
	Normalized HCMV PC binding FcγRIIIa	NE-T to NE-NT	Wilcoxon rank sum	0.2	0.5
	Normalized HCMV gHgLgO binding FcγRIIIa	NE-T to NE-NT	Wilcoxon rank sum	>0.9	>0.9
	Normalized HSV gD-1 binding FcγRIIIa	NE-T to NE-NT	Wilcoxon rank sum	0.3	0.5
	Normalized HSV gB binding FcγRIIIa	NE-T to NE-NT	Wilcoxon rank sum	0.4	0.7
Figure 4D	Normalized HCMV gB binding with FcγRN	M-T to M-NT	Wilcoxon rank sum	0.12	0.5
	Normalized HCMV PC binding FcγRN	M-T to M-NT	Wilcoxon rank sum	0.8	>0.9
	Normalized HCMV gHgLgO binding FcγRN	M-T to M-NT	Wilcoxon rank sum	>0.9	>0.9
	Normalized HSV gD-1 binding FcγRN	M-T to M-NT	Wilcoxon rank sum	0.4	0.5
	Normalized HSV gB binding FcγRN	M-T to M-NT	Wilcoxon rank sum	0.5	0.6
	Normalized HCMV gB binding with FcγRN	NE-T to NE-NT	Wilcoxon rank sum	0.083	0.5
	Normalized HCMV PC binding FcγRN	NE-T to NE-NT	Wilcoxon rank sum	0.2	0.5
	Normalized HCMV gHgLgO binding FcγRN	NE-T to NE-NT	Wilcoxon rank sum	0.2	0.5
	Normalized HSV gD-1 binding FcγRN	NE-T to NE-NT	Wilcoxon rank sum	0.2	0.5
	Normalized HSV gB binding FcγRN	NE-T to NE-NT	Wilcoxon rank sum	0.3	0.5
